# Consumption of ultra-processed foods and female infertility: a cross-sectional study

**DOI:** 10.3389/fpubh.2025.1597910

**Published:** 2025-06-30

**Authors:** Adam T. Evans, Snigdha Alur-Gupta, Euridice Martinez Steele, Ying Meng, Alex J. Knutson, Wendy S. Vitek

**Affiliations:** ^1^Department of Obstetrics and Gynecology, University of Rochester Medical Center, Rochester, NY, United States; ^2^Department of Nutrition, School of Public Health, University of São Paulo, São Paulo, Brazil; ^3^Center for Epidemiological Studies in Health and Nutrition, University of São Paulo, São Paulo, Brazil; ^4^School of Nursing, University of Rochester, Rochester, NY, United States

**Keywords:** ultra-processed foods, infertility, Nova system, NHANES, dietary intake, nutrition

## Abstract

**Background:**

A high dietary consumption of ultra-processed foods (UPF) has been associated with abnormal semen analysis parameters in males; however, it is unclear whether there is an impact on female reproduction. The objective of this study is to assess whether high consumption of UPF is associated with female infertility.

**Methods:**

A cross-sectional analysis of females aged 18–45 years who participated in the National Health and Nutrition Examination Survey (NHANES) from 2013 to 2018 was performed. Females were eligible for inclusion if they provided a 24-h dietary recall and responded to a question regarding infertility. Foods reported during a 24-h dietary recall were classified according to the Nova system. The relative percentage of total energy consumed from UPF and unprocessed/minimally processed foods within a 24-h period was calculated.

**Results:**

The prevalence of infertility was 11%. The overall mean percentage of daily UPF and unprocessed/minimally processed food intake among respondents was 57 and 29%, respectively. There was no difference in the odds of female infertility when comparing females in the lowest tertile of UPF consumption to peers in the middle [aOR = 1.37, 95% CI (0.96, 1.95)] or highest tertile of consumption [aOR = 1.26, 95% CI (0.91, 1.74)]. Similarly, there was no difference in the odds of infertility between participants in the middle [aOR = 1.39, 95% CI (0.83, 2.33)] or highest tertile [aOR = 0.73, 95% CI (0.34, 1.54)] of unprocessed/minimally processed food consumption relative to those in the lowest tertile. In an intermediate model removing body mass index (BMI) as a covariate, an increased odds of infertility was noted among females with the highest tertile of UPF consumption relative to those in the lowest tertile [aOR = 1.46, 95% CI (1.01, 2.09)].

**Conclusion:**

Among women of reproductive age, high consumption of UPF is associated with female infertility. BMI is a mediator of the association between UPF and female infertility.

## Introduction

Dietary trends within the United States demonstrate a steady increase in the consumption of ultra-processed foods (UPF) (1, 2). While various systems exist to classify foods according to their degree of industrial processing, the Nova system, established in 2009, has been widely adopted in the realms of both research and food policy (3–5). The Nova system classifies foods and beverages into one of four groups based on the methods and techniques utilized to produce a given commercial product (6, 7). Specifically, UPF refer to industrial preparations of foodstuffs containing little to no whole foods which are combined with various cosmetic additives and substances of rare culinary use to create ready-to-eat/heat meals (6–8). Among adults in the United States participating in the National Health and Nutrition Examination Survey (NHANES), the percentage of daily calories derived from UPF increased from 53.5 to 57% between 2001 and 2018 along with an associated decline in consumption of unprocessed/minimally processed foods (2). Similar trends have also been seen within NHANES in an adolescent population in the United States, demonstrating that UPF represent the majority of total daily energy intake for both individuals and families (1).

High consumption of UPF has been linked to obesity, cardiovascular disease, metabolic dysfunction, increased all-cause mortality and may play a role in gynecologic disease (9–11). Significant intake of UPF has been associated with an increased incidence of ovarian cancer and subsequent cancer-related mortality (10). Given these trends with respect to ovarian pathology, it is necessary to explore the potential impact of UPF consumption on ovarian function and female reproduction. Further, UPF consumption may be linked to infertility; it has been demonstrated that males with high dietary intake of UPF have increased odds of asthenozoospermia (12). However, it has not yet been explored whether UPF consumption may also contribute to an increased risk of female infertility. Furthermore, it appears that the relationship between body mass index (BMI) and consumption of UPF may be modified by age (13). Given that population-based studies have shown UPF represent greater than 50% of calories consumed by adults in the United States and framed within the context of the literature, it is critical to understand whether these dietary patterns may influence an individual’s ability to conceive (1, 2). This question models an effort to understand the impact of UPF on human health and disease, expressed by the United States Department of Agriculture (USDA) prior to issuing the Dietary Guidelines for Americans, 2025–2030 (14). Female infertility is defined clinically based on patient history of 12 months of attempting to become pregnant without success (15). An understanding of the potential impact of UPF on female reproduction is necessary to ensure appropriate targeting of public health initiatives, allocation of community resources, patient counseling, and subsequent therapeutic interventions for females and couples seeking pregnancy.

We hypothesize that among females of reproductive age, individuals with higher daily consumption of UPF will have a higher rate of infertility than peers with lower dietary intake of these foods. Therefore, the objective of this study is to examine the association between UPF consumption and female infertility among a cohort of reproductive-aged females participating in NHANES from 2013 to 2018.

## Methods

### Study design

A cross-sectional study was performed utilizing data from NHANES from 2013 to 2018. Survey data was obtained from non-institutionalized adults in the United States and derived from a combination of in-person interviews, clinical assessments, and laboratory evaluation (16, 17). Data was collected in 2-year increments by the National Center for Health Statistics (NCHS), employing intentional oversampling of specific populations and analytical weighting to reflect health and demographic trends of the United States (17, 18). Each NHANES survey and study was approved by the NCHS Ethics Review Board. Questions regarding infertility were integrated into the NHANES questionnaire in 2013. As a result, NHANES surveys from 2013 to 2018 were utilized for this study. Given use of publicly available, de-identified data in this study, local Institutional Review Board approval was not indicated.

### Study population

Females between the ages of 18–45 years old, with a valid day 1 dietary recall, and self-reported infertility status were eligible for study inclusion. The range of 18–45 years was chosen to reflect the population of women of reproductive age who may be attempting to become pregnant. The decision was made to end data collection at age 45 as this is the age where fertility treatments are typically no longer offered due to low success rates and the age-related decline in ovarian reserve.

### Exposure assessment

Dietary intake was assessed from the first of two 24-h dietary recalls conducted by trained interviewers. The NHANES analytical guidelines and the literature note that a single 24-h dietary recall is sufficient when estimating the mean usual intake of an individual as within-person random error caused by daily variation tends to average out and does not bias the mean (19). Reported foods were classified according to the level of processing using the Nova classification system as previously described (7, 8). The Nova classification system categorizes food into four groups based on their industrial processing level and purpose. Unprocessed or minimally processed foods are subjected to little or no processing techniques without the addition of processed culinary ingredients. Processed culinary ingredients are exacted from unprocessed foods (sugar or oils) or nature (salt) and used for culinary preparations. Processed foods are industrially manufactured from unprocessed/minimally processed foods combined with processed culinary ingredients. UPF are defined as foods and beverages that have undergone multiple industrial processes, often containing substances of rare culinary use and/or cosmetic additives (8).

The relative percentage of total energy derived from UPF within a 24-h period served as the primary predictor. The relative percentage of total daily energy intake from unprocessed/minimally processed foods served as the secondary predictor. To facilitate comparison, food consumption data for UPF and unprocessed/minimally processed foods was categorized into tertiles.

### Outcomes

The primary outcome was the rate of self-reported infertility. Infertility status was determined by patient self-report to survey question RHQ074: “have you ever attempted to become pregnant over a period of at least a year without becoming pregnant?” Participants with an affirmative response were classified as having infertility (15, 20).

### Covariates

Covariates adjusted for in the analysis included: age, BMI underweight (BMI < 18.5 kg/m^2^), normal weight (18.5–24.9 kg/m^2^), overweight (25–29.9 kg/m^2^), class I obesity (30–34.9 kg/m^2^), class II obesity (35–39.9 kg/m^2^), class III obesity (>40 kg/m^2^), marital status (married/living with partner, widowed/divorced/separated, and never married), and self-reported race/ethnicity (non-Hispanic white, non-Hispanic black, Hispanic, Asian, and other). These variables were chosen *a priori* due to their potential impact on both self-reported infertility and nutrition (20–26). Due to a high percentage of missing data, tobacco and alcohol use were unable to be included as covariates (20).

### Statistical Methods

Statistical analysis and sample weighting were performed in accordance with the NHANES analytic guidelines (18). Group comparisons were performed using chi square tests per NHANES guidelines. Statistical significance was set at *p* < 0.05. To assess for associations between total energy consumed from a given Nova group and infertility, weighted univariate followed by multivariable logistic regression was performed accounting for the noted covariates to assess for adjusted odds ratios with 95% confidence intervals. Given the prior findings in the literature that age may modify the relationship between BMI and UPF consumption, an interaction between BMI and age was also assessed in our study population by utilizing weighted multivariable logistic regression models (13). Analysis was performed using Stata 18 (College Station, TX).

## Results

A total of 3,060 respondents met inclusion criteria, which represented 58,112,352 females with NHANES survey weighting. The prevalence of infertility in the surveyed population was 11%. Females with self-reported infertility had a higher mean age (35.2 vs. 30.9 years), were more likely to be obese (55.9% vs. 37.7%), had a higher mean BMI (32.5 kg/m2 vs. 28.9 kg/m2), and were more likely to be married or living with a partner (76.8% vs. 58.1%) relative to females without infertility (Table 1). Among all respondents, the mean percentage of daily energy intake from UPF and unprocessed/minimally processed foods was 57 and 29%, respectively. Females with self-reported infertility had higher mean consumption of UPF (57.5% vs. 56.0%) and lower consumption of unprocessed/minimally processed foods (28.1% vs. 29.4%) than females without infertility however, these did not reach statistical significance (Table 1).

**Table 1:** Demographics of NHANES respondents, aged 18–45 years.

Characteristic	Without infertility *N* = 51,914,845	Self-reported infertility *N* = 6,197,507	*p*-value
	% or mean (SE)	% or mean (SE)	
Age*^a^* (y)	30.9 (0.2)	35.2 (0.5)	<0.01
BMI*^a^* (kg/m^2^)	28.9 (0.2)	32.5 (0.8)	<0.01
Underweight	2.3 (0.3)	1.5 (0.6)	
Normal weight	34.9 (1.3)	25.0 (3.4)	
Overweight	25.7 (0.8)	17.6 (2.7)	<0.01
Class I obesity	17.2 (1.1)	20.7 (2.5)	
Class II obesity	9.8 (0.8)	15.9 (2.3)	
Class III obesity	10.1 (0.7)	19.3 (3.0)	
Race/Ethnicity, %			
Non-Hispanic white	53.9 (2.5)	58.6 (4.1)	0.50
Non-Hispanic black	14.1 (1.5)	13.2 (1.9)	
Asian	6.7 (0.7)	5.6 (1.1)	
Hispanic	21.1 (1.7)	17.7 (3.0)	
Other	4.1 (0.5)	5.0 (1.6)	
Daily UPF intake, %			
Mean	56.0 (0.5)	57.5 (1.1)	0.20
Tertile 1	35.5 (5.2)	35.7 (1.2)	0.91
Tertile 2	57.8 (1.9)	58.2 (5.0)	0.43
Tertile 3	77.7 (3.6)	76.8 (9.9)	0.37
Daily unprocessed/minimally processed food intake, %			
Mean	29.4 (0.8)	28.1 (1.7)	0.51
Tertile 1	13.3 (0.3)	15.4 (0.7)	<0.001
Tertile 2	28.9 (0.2)	28.0 (0.6)	0.26
Tertile 3	50.2 (0.9)	48.5 (2.0)	0.48

Data are expressed as a percentage with linearized SE unless noted otherwise. ^a^Mean.

To facilitate comparison, the mean percentage of daily UPF and unprocessed/minimally processed food consumption was divided into tertiles for both females without infertility and for individuals with self-reported infertility (Table 1). There was no difference in the odds of infertility when comparing females in the middle [aOR = 1.37, 95% CI (0.96, 1.95)] or highest [aOR = 1.26, 95% CI (0.91, 1.74)] tertile of UPF consumption with those in the lowest after adjusting for covariates (Table 2). Further, there was no association between higher consumption of unprocessed/minimally processed foods and infertility status relative to females in the lowest tertile of daily consumption [middle tertile: aOR = 1.39, 95% CI (0.83, 2.33), highest tertile: aOR = 0.73, 95% CI (0.34, 1.54)] (Table 3). When assessing for interaction between age and BMI, a significant interaction was noted only in individuals with class III obesity between the ages of 38–39 years although with a small magnitude [aOR = 0.14, 95% CI (0.02, 0.78)]. Given the absence of a dose response, it is possible this is due to chance. In addition, when age and BMI were analyzed as continuous variables the interaction term was not significant. Given BMI could act as both a mediator and confounder in the association between UPF and infertility an intermediate model was run without adjustment for BMI which noted an increased odds of infertility among females with the highest tertile of UPF consumption relative to those in the lowest tertile [aOR = 1.46, 95% CI (1.01, 2.09)] (Table 2).

**Table 2:** Relationship between daily consumption of UPF (by tertile) and infertility.

Tertile of consumption (% of daily energy intake)	OR (95% CI)	aOR* (95% CI)	aOR** (95% CI)
T1	Reference	Reference	Reference
T2	1.35 (0.96, 1.88)	1.37 (0.96, 1.95)	1.44 (0.99, 2.09)
T3	1.26 (0.91, 1.75)	1.26 (0.91, 1.74)	**1.46 (1.01, 2.09)**

*Results are derived from a weighted multivariable logistic regression adjusting for age, marital status, income and BMI. **Results are derived from a weighted multivariable logistic regression with BMI removed, therefore adjusting for age, marital status, and income. T1, lowest tertile of consumption; T2, middle tertile of consumption; T3, highest tertile of consumption. Bold indicates statistical significance.

**Table 3:** Relationship between daily consumption of unprocessed/minimally processed foods (by tertile) and infertility.

Tertile of consumption (% of daily energy intake)	OR (95% CI)	aOR* (95% CI)	aOR** (95% CI)
T1	Reference	Reference	Reference
T2	1.55 (0.85, 2.82)	1.39 (0.83, 2.33)	1.23 (0.69, 2.17)
T3	0.79 (0.36, 1.75)	0.73 (0.34, 1.54)	0.66 (0.28, 1.53)

*Results are derived from a weighted multivariable logistic regression adjusting for age, marital status, income and BMI. **Results are derived from a weighted multivariable logistic regression with BMI removed, therefore adjusting for age, marital status, and income. T1, lowest tertile of consumption; T2, middle tertile of consumption; T3, highest tertile of consumption.

## Discussion

In this cross-sectional study of females of reproductive age participating in NHANES between 2013 and 2018, higher consumption of UPF is associated with increased odds of female infertility and BMI is a mediator (27). Moreover, higher consumption of unprocessed/minimally processed foods was not associated with reduced odds of infertility in this population.

This represents the first investigation into the potential impact of UPF consumption on female infertility in the United States. An association between increased consumption of trans-fatty acids and the risk of anovulatory infertility has been previously documented, but the role of UPF has not been explored (28). We demonstrated that while there was no increase in the odds of female infertility among individuals with higher consumption of UPF when adjusting for all covariates, this relationship changed when BMI was removed from the model. Given the possible collinearity between BMI and UPF consumption—and therefore the potential for this variable to act as both a confounder and a mediator—an intermediate model was run without BMI as a covariate. These findings noted an increase in the odds of female infertility among participants in the highest tertile of UPF consumption but no changes in any other groups of participants. Therefore, it appears that BMI may be a mediator of the association between UPF and female infertility.

A prior examination of UPF consumption within a prospective cohort of Chinese men treated at infertility clinics reported an increased odds of asthenozoospermia in individuals with higher percentages of daily UPF intake (12). While we did not demonstrate a similar trend with respect to self-reported female infertility when adjusting for BMI in our model [as was done by Lv et al. (12)], an increase was noted in our intermediate model (removal of BMI) among participants in the highest tertile of UPF consumption. When comparing the results of our study with those of Lv et al. (12), it is important to note that there are some key differences between our populations. First, there are inherent differences in the regional diets of China and the United States both in terms of food offerings and meal structure/composition. This point is further underscored by the differences in tertiles of UPF consumption within our respective study populations. Individuals in the highest tertile of UPF consumption within the Chinese cohort (>15.12%) had lower intake than females in the lowest and middle tertile of our NHANES cohort. Furthermore, the mean percentage of daily UPF consumption within our study population was greater than 56% for both females with self-reported infertility and females without infertility, underscoring critical differences in the nutrition patterns of our participants. It remains unclear whether there is a “threshold” of UPF consumption necessary to facilitate reproductive dysfunction and whether this may vary among different cultures/populations. Further prospective studies would be necessary to delineate whether such a threshold exists, to allow for better targeting of food and nutrition campaigns.

While a prior study demonstrated that higher UPF consumption increased the odds of ovarian cancer, based on the findings of our study, it is unclear if this dietary pattern leads to ovarian dysfunction (10). Data specific to menstrual cyclicity or ovarian reserve is not collected by NHANES and therefore is unable to be analyzed in this study. Further, due to limitations of the NHANES data we are unable to determine the type of infertility experienced by respondents. Therefore, we are unable to assess whether high UPF consumption may be associated with specific etiologies of female infertility such as oligo-anovulation. Beyond this, it is unclear how UPF consumption may impact follicular or luteal phase dynamics in females of reproductive age. Further prospective exploration of these questions is necessary to better delineate the impact of UPF on menstrual cycle dynamics, ovarian reserve, ovulation, and infertility.

The use of NHANES is a strength of our study as sample weighting provides significant power to detect an association between UPF consumption and self-reported infertility. Beyond this, due to sampling methodology of NHANES, the data reflect the demographic trends of the United States therefore leading to good generalizability of our findings. Additionally, we utilized the Nova system to describe the degree of food processing which has been widely utilized in the literature (Table 4). Specific classification of foods consumed by participants and calculation of the relative percentage of daily energy derived from these foods was performed utilizing the standardized methodology previously reported by Steele et al. (7). A rigorous definition of infertility was utilized (at least 12 months of unprotected intercourse without achieving a pregnancy). However, it is important to note that this may lead to underreporting of infertility among individuals above the age of 35 years, who based on American Society for Reproductive Medicine (ASRM) guidelines would meet the definition of infertility after at least 6 months of attempting pregnancy without success but have not yet reached one year (15). Therefore, these individuals may have been included in the group without infertility which could bias results toward the null. Furthermore, lack of detail on branding and degree of processing of foods may lead to Nova misclassification which therefore could dilute the studied associations toward to null. Moreover, due to social stigma participants may have been less likely to disclose their infertility status in NHANES which may further impact our results. Consistent with NHANES guidelines, a single 24-h dietary recall was used in our study which has shown to be sufficient for estimation of an individual’s mean usual intake as day-to-day variation averages out and does not bias the mean (19). While some degree of measurement error is inherent in dietary recall data, this approach is unlikely to introduce systematic bias in estimating population-level associations. However, we acknowledge this as a possible limitation in our methodology as this may not accurately capture usual UPF intake and therefore further studies incorporating multiple dietary recalls would strengthen our findings. Due to limitation in the NHANES data collection we are unable to ascertain the age at which an individual experienced infertility. We are only able to note the age at which they completed the NHANES survey. As a result, we cannot directly assess the association of UPF at the time of infertility. However, we did assess for any impact of age as an effect modifier of the association between UPF and infertility and did not find a statistically significant interaction. Given the age-related decline in ovarian reserve, further studies exploring this are necessary to elucidate any possible relationship. With respect to UPF exposure, it is plausible that there may be a “critical window” at which time high consumption of these foods may impact an individual’s fertility most. Is it therefore possible that if such a window exists in youth that current eating behaviors or UPF exposure may be underestimating the true impact of consumption observed in our study. Further longitudinal studies examining the long-term impact of UPF consumption in childhood and adolescence on fertility outcomes are necessary to better delineate these relationships.

**Table 4:** Summary of Nova system of food classification.

Nova category	Definition (7, 8)	Foods (7)
Group 1: unprocessed/minimally processed foods	Foods that are subjected to little or no processing techniques without the addition of processed culinary ingredients	Fresh or frozen fruits and vegetables Eggs Raw meats and fish Pasteurized milk Unsalted/unseasoned nuts Legumes
Group 2: processed culinary ingredients	Ingredients extracted from unprocessed foods or nature and used for culinary preparations	Sugar Salt Plant oils Butter
Group 3: processed foods	Foods that are industrially manufactured from unprocessed/minimally processed foods combined with processed culinary ingredients	Salted nuts Cheese Breads made with flour, water, and yeast Canned vegetables, legumes and fruits Wine, beer Cured, salted or smoked meats
Group 4: ultra-processed foods	Foods and beverages that have undergone multiple industrial processes, often containing substances of rare culinary use and/or cosmetic additives	Sweetened breakfast cereals Frozen read to eat/heat meals (chicken nuggets) Prepackaged sweet or savory snack foods Sodas, sweetened beverages Instant soups Candy

This study determined that among a large cohort of reproductive aged females in the United States, individuals with the highest tertile of UPF consumption had an increased odds of infertility and that this association appears to be modified by BMI. Due to the high prevalence of UPF in the diet of individuals in the United States additional prospective studies incorporating diets with lower percentages of UPF are necessary to validate these findings and to determine whether a “threshold” of reproductive dysfunction exists.

## Data Availability

Publicly available datasets were analyzed in this study. This data can be found at: https://wwwn.cdc.gov/nchs/nhanes/.
